# Modulatory effect of levodopa on the basal ganglia-cerebellum connectivity in Parkinson’s disease

**DOI:** 10.1038/s41531-025-00954-9

**Published:** 2025-05-06

**Authors:** Juyoung Jenna Yun, Subati Abulikemu, Kodchakorn Love Jangwanich, Yen F. Tai, Shlomi Haar

**Affiliations:** 1 Department of Brain Sciences, Imperial College London, London, UK; 2 UK Dementia Research Institute – Care Research and Technology Centre, Imperial College London, London, UK; 3 Department of Neurology, Charing Cross Hospital, London, UK

**Keywords:** Parkinson's disease, Parkinson's disease, Basal ganglia, Cerebellum

## Abstract

Long-term levodopa use in Parkinson’s disease is associated with declining efficacy and motor complications. Understanding its effects on brain reorganisation is vital for optimizing therapy. Using data from Parkinson’s Progression Marker Initiative, we investigated levodopa’s impact on resting-state functional connectivity within the cortico-basal ganglia-cerebellum system in 29 patients, under drug-naïve and levodopa-medicated conditions. Univariate comparisons of inter-regional connectivity between conditions were conducted, and multivariate combinations of connections within and between networks were assessed. No significant effect of levodopa was found using the univariate seed-based approach. However, the network connectivity pattern between basal ganglia and cerebellum showed robust classification power. Eigenvector Centrality Mapping (ECM) further identified functional hubs, with cerebellar hubs being the only ones within the cortico-basal ganglia-cerebellum system affected by medication. Our study provides further insight into the importance of inter-network functional connectivity in Parkinson’s and the impact of levodopa medication, highlighting the often-overlooked role of the cerebellum.

## Introduction

Parkinson’s disease (PD) is the second most prevalent neurodegenerative disorder affecting 1–2% of the population over 50 years of age^1^. Clinically, PD is a movement disorder characterised by bradykinesia, resting tremor, rigidity, and postural instability^1,2^. The cardinal pathology of PD is the depletion of nigrostriatal dopaminergic (DA) neurons with consequent dysfunction of cortico-striatal-thalamic-cortical circuits^3,4^ (CSTC). Since its development in the late 1960s, levodopa (L-3,4-dihydroxyphenylalanine), as a direct precursor to DA, has remained the most efficacious symptomatic therapy for PD^5^. With prolonged treatment, however, the clinical response to levodopa progressively declines (i.e., wearing-off phenomenon) and leads to the occurrence of motor complications, including fluctuations, dyskinesia, and dystonia^6,7^. Hence, it is crucial to uncover levodopa-induced neural effects and functional brain reorganisation to provide new insights into optimising PD therapeutics.

Resting-state functional connectivity (rs-FC) has been increasingly examined in PD brains and their modulations following levodopa medication, in accordance with the nexopathy framework conceptualising neurodegenerative diseases as disconnection syndromes^8^. Aberrant functional integration between cortical sensorimotor areas and striatum was consistently detected and deemed a fundamental pathological remapping in PD^9–11^. Studies show that levodopa affects connectivity in the basal ganglia (BG)-thalamic-motor cortical system, but findings are conflicted, with both increases and decreases in connectivity observed^11^. This variability may be due to differences in study methods, patient conditions, and the choice of regions analysed. Past reports were often based on the acute levodopa responses, and longitudinal rs-FC changes following stable levodopa medication in drug-naïve patients are yet to be extensively investigated.

Apart from the conventional CSTC circuit dysfunction in PD, the role of the cerebellum has been emphasised. The cerebellum is likely to exert both crucial pathological and compensatory effects in PD^12,13^. Relative to healthy controls, PD patients displayed weakened striatum-cerebellum connectivity, significantly greater activations of the bilateral cerebellum, and strengthened functional coupling between the cerebellum and the cortical motor network^14–16^. The diminished FC might reflect the aberrant BG signalling over the cerebellum in PD, whereas the cerebellar-thalamic-cortical circuit was thought to be increasingly strengthened as the pathology progresses to preserve motor functions^12,17^. Acute levodopa administration was shown to substantially elevate the cerebellum connectivity to subcortical regions of the motor system, including the thalamus, putamen, globus pallidus (GP), and brainstem^7^. A recent consensus paper emphasised the role of the cerebellum within the integrated cortico-BG (-thalamo)-cerebellum system where functional and plasticity processes of the networks were interactive^18^. A system-level mechanism of cerebellum, cortex, and BG in Parkinson’s and its plastic changes following levodopa medication is yet to be further examined.

In this project, we adopted a longitudinal design to investigate the effect of levodopa on motor-functional reorganisation in PD under stable levodopa treatment. This included a PD cohort who transitioned from drug-naïve to levodopa-medicated states. We investigated regional and network connectivity in the motor cortex, BG, and cerebellum employing respective seed- and network-level analyses. Our approach enabled the assessment of levodopa modulation at varying neural levels and the comparisons of which under different research designs.

## Results

### Sample selection

**Fig. 1:**
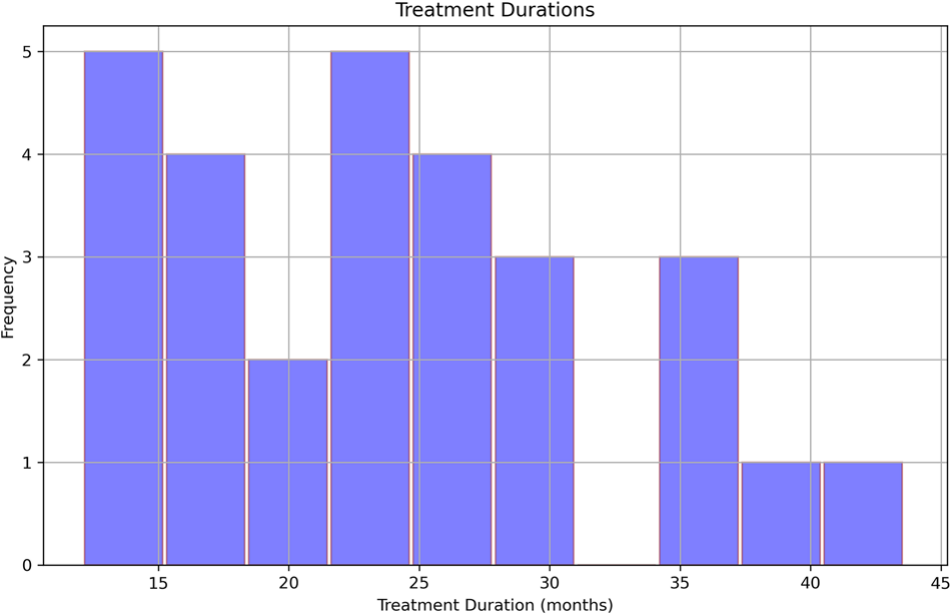
Sample selection based on treatment and disease durations. Treatment durations at selected levodopa scans (*median* = 24 months; *IQR* = 10.9 months; *maximum* = 44 months; *minimum* = 12 months).

The longitudinal cohort consisted of 29 patients who had resting-state scans at both de novo and levodopa-medicated states (Table 1). Given that all patients had multiple scans while on levodopa medication, we required a minimum of 12 months on medication and then evaluated all possible medicated scans and computed the variance of treatment durations for each combination. We selected the scans that minimised this variance, ensuring treatment durations across the cohort were as similar as possible (Fig. 1).

**Table 1:** Demographics and clinical measures of patients in the longitudinal dataset

	De novo	Levodopa	*P* Values
Age (years)	59.65 (17.25)	61.6 (17)	***p*** < **0.001**
Gender (male/total)	18/29	18/29	
Disease Duration (months)	15.22 (7.58)	51.73 (2.11)	***p*** < **0.001**
Treatment Duration (months)		23.85 (10.9)	-
Levodopa equivalent daily dose (mg)		153.5 (200)	-
Clinical measures	ON-levodopa: *n* = 29		
Hoehn and Yahr scale	2 (1)	2 (0)	0.18
UPDRS-I	1 (2)	1 (1)	0.5
UPDRS-II	6 (4.75)	6 (6)	0.66
UPDRS-III	17.5 (10.75)	15(14)	0.22
Tremor subscale	1 (2)	0 (0)	**0.0054**
Rigidity subscale	5 (3)	3 (4)	0.37
Gait subscale	1 (1)	0 (1)	0.45
MoCA	28 (2)	29 (1)	0.32

Demographics and clinical measures of study participants. Values are medians (interquartile range). For gender, values are the numbers of males [% of males]. For patients who initiated levodopa treatment, the OFF levodopa was defined as not having taken medication for at least 6 h. We performed paired-sample comparisons of the UPDRS measures between the de novo state ON state. Only ON medication scores were included as only a few levodopa-medicated patients had UPDRS scores in their OFF state. MoCA score of 1 and UPDRS III scores of 3 patients were missing. Due to the non-Gaussian distributions of the variables, we conducted non-parametric Wilcoxon signed-rank and rank-sum tests for within- and between-group comparisons, respectively.

Statistical significance, *p* < 0.05.

Table 1 provides the demographic and clinical descriptors of the selected patients. The median interval between two visits was 39 months (*IQR* = 10 months). At time point two, ON-levodopa patients (within 6 h after the last levodopa intake) experienced a slight attenuation of motor symptoms measured by part three of the Movement Disorder Society-Unified PD Rating Scale^19^ (UPDRS-III) but with no significant difference (*median of paired difference* = 3, *IQR* = 3.75, *p* = 0.25). UPDRS-III scores after the medication wears off (over 6 h after the last levodopa intake) were not recorded for most participants.

### Functional connectivity strength mapping

**Fig. 2:**
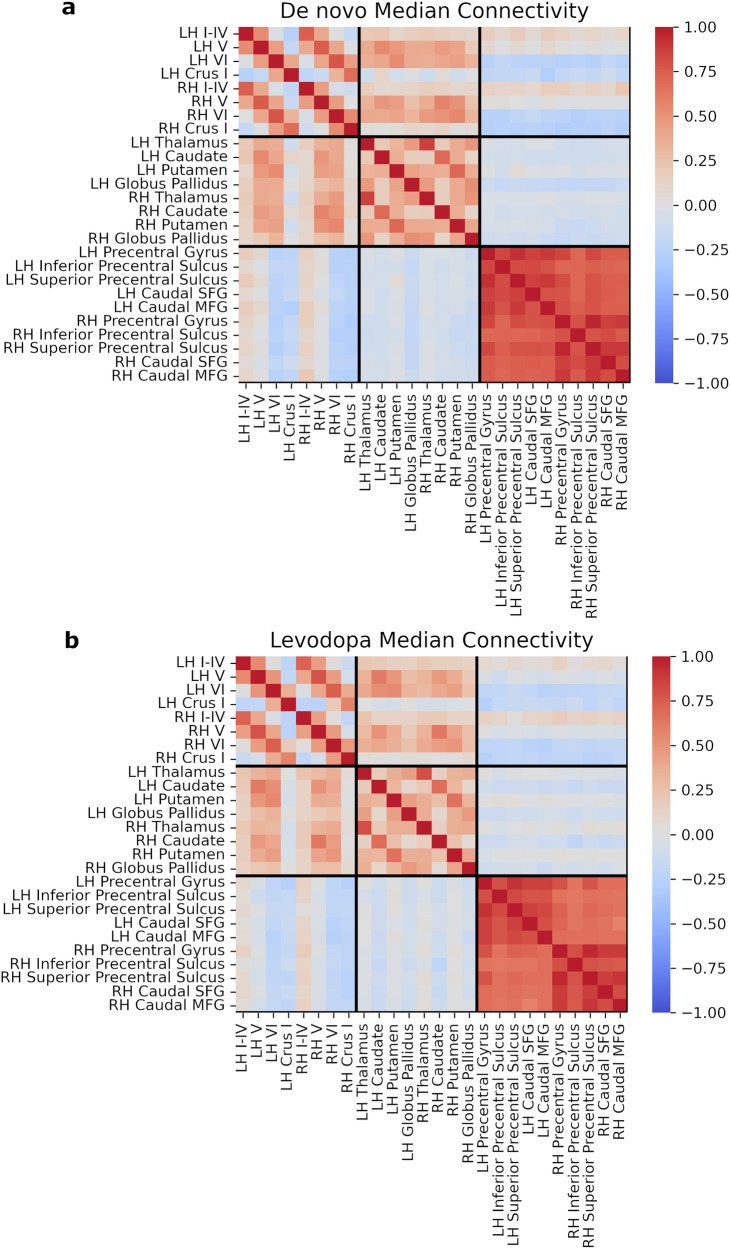
Interregional functional connectivity strength. Connectivity matrix of the median interregional rs-FC for the de novo (**a**) and levodopa medication (**b**) states. Regions are organised in the order of cerebellar, basal ganglia (and thalamus), and motor cortical regions. LH left hemisphere, RH right hemisphere, MFG middle frontal gyrus, SFG superior frontal gyrus.

The general connectivity patterns (Fig. 2) across the subregions of cortical motor areas, BG, and cerebellum (listed in Table 2) were similar between de novo and levodopa states. Strong interconnectedness within the cortical motor network, moderate connections within the cerebellum as well as within BG and thalamus, and limited cross-network coherence were commonly manifested. In examining the regional connectivity within the cerebellum and basal ganglia a strong interhemispheric connectivity pattern is observed. The overall connectivity pattern seems very stable and consistent across the medication states (Fig. 2).

**Table 2:** Selected ROIs. Subcortical ROIs are voxel-based, whereas motor cortical ROIs are vertex-based

Cerebellar ROIs (voxels)
Cerebellar lobules I–IV
Cerebellar lobule V
Cerebellar lobule VI
Cerebellar Crus I
**Thalamoganglionic ROIs (voxels)**
Thalamus (Tha)
Caudate nucleus (Cdn)
Putamen (Pu)
Globus pallidum (GP)
**Motor cortical ROIs (vertices)**
Precentral gyrus (PreCG)
Inferior precentral sulcus (InfPreCS)
Superior precentral sulcus (SupPreCS)
Caudal superior frontal gyrus (SFGcau)
Caudal middle frontal gyrus (MFGcau)

### Within-group comparison of connectivity strength

**Fig. 3:**
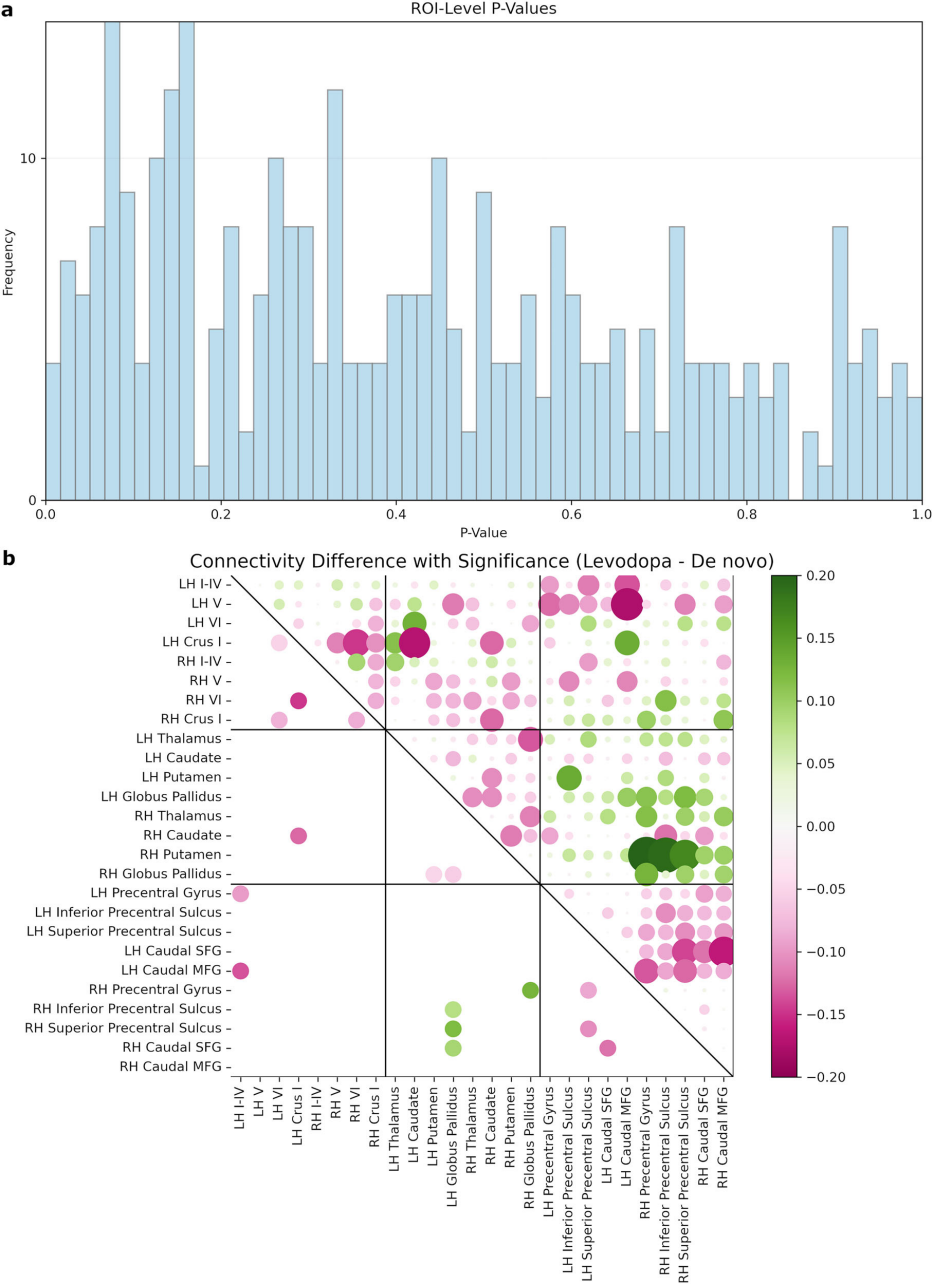
Within-group comparisons in region-of-interest analysis. **a** Frequency histograms of *p*-values in region-of-interest analyses. **b** *Upper triangle:* Median differences of connectivity magnitude between levodopa and de novo states in longitudinal analysis; the size of the circles is proportional to the absolute values. *Lower triangle:* Significant within-participant connectivity contrasts at *p* < 0.05 before FDR correction (green circles represent the levodopa connections greater than de novo, and pink circles depict more intense de novo connections relative to levodopa).

We first statistically assessed the univariate effect of levodopa on each interregional functional coupling across the three networks. The distribution of *p* values in within-participant comparisons of connectivity strength was right-skewed (Fig. 3a) and displayed a meaningful trend towards significance. Before correcting for the multiple comparisons, the univariate tests showed higher functional synchrony at the drug-naïve state for multiple connections within-cerebellum, within-BG, within-motor cortex, and some cerebellar connections with the BG and cortex. Some BG-cortex connections showed stronger connectivity at the medicated state (Fig. 3b). Nevertheless, no region-of-interest (ROI)-level comparisons survived false discovery rate (FDR) correction.

### Network level differences in functional connectivity

To test the potential effect of levodopa medication on functional connectivity on a network level, i.e., beyond individual ROI-to-ROI connections, we used a support vector machine (SVM) to classify the patterns of connectivity in the network. To assess meaningful changes within and between regions of the motor control circuitry instead of applying a single graph model on the entire network we considered three subnetworks of the motor CSTC circuitry (cortical motor areas, BG, and cerebellum) and classified the medication conditions separately based on the connections within each one of them and between each pair.

**Fig. 4:**
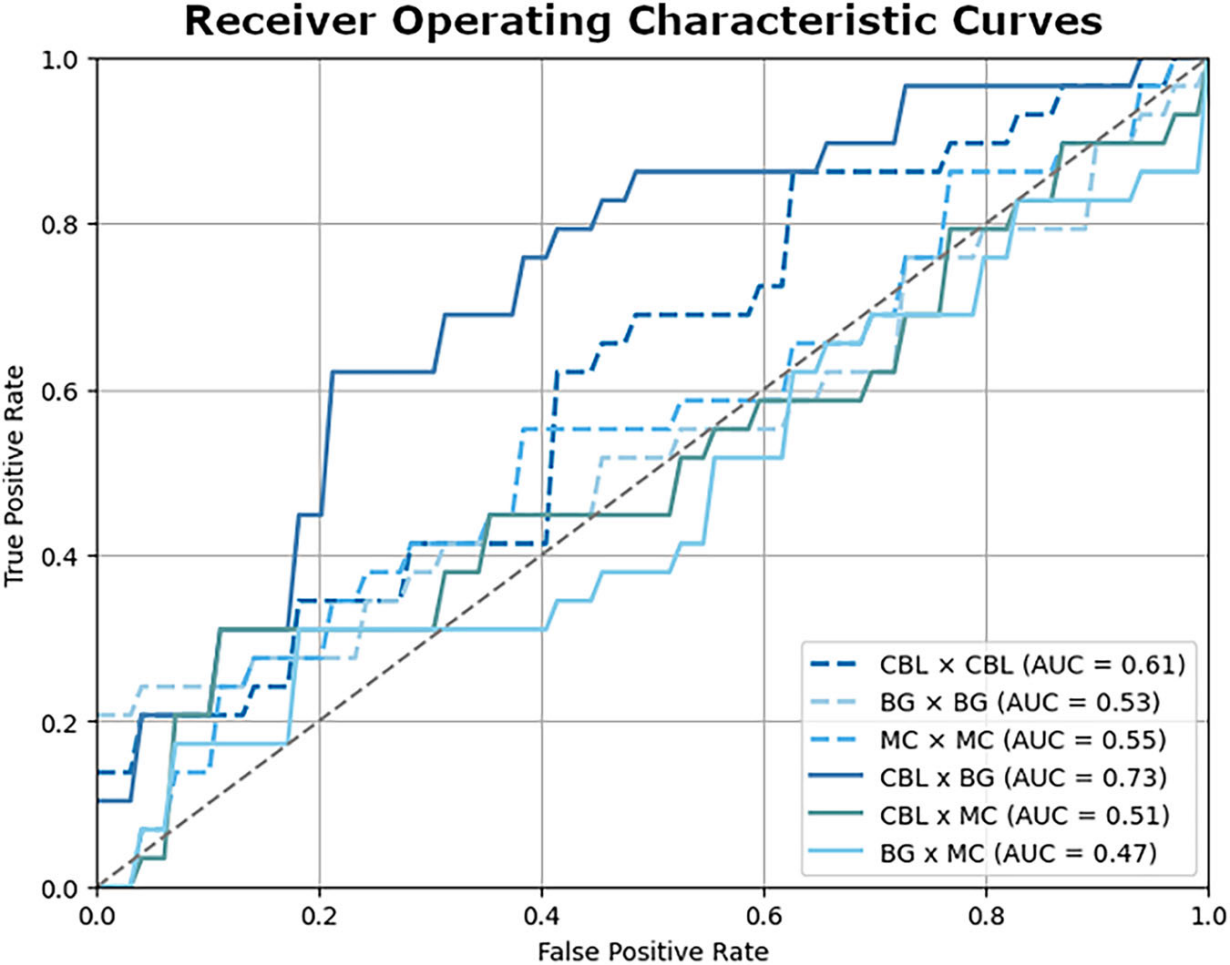
Receiver operating characteristic curve and area under the curve. The receiver operating characteristic curves were constructed for the support vector machine using the different subnetworks. Between subnetworks connections are in solid lines and within subnetworks are in dashed lines. CBL cerebellum, BG basal ganglia, MC motor cortex.

The linear SVM implemented with the BG-cerebellum feature set yielded the highest discriminating efficacy in both datasets (Fig. 4 & Table 3). The model had an *area under the receiver operating characteristic curve (AUC)* of 0.73 and reached an overall *accuracy* of 69%, accurately classifying 17 out of 29 levodopa cases (*sensitivity* = 0.59) and 23 out of 29 drug-naïve states (*specificity* = 0.79). The permutation test showed that the accuracy was significantly higher than chance (*p* < 0.001), and the significance was retained after adjusting for multiple testing (*FDR-adjusted p* < 0.001). The only other significant model was within the motor cortex features, with an *accuracy* of 57% sensitivity of 0.34, a specificity of 0.79, and an *AUC* of 0.55 (uncorrected *p* = 0.028). However, its statistical significance did not survive FDR correction (FDR-adjusted *p* = 0.085). This implies that only the BG-cerebellum network had robust information to effectively distinguish treatment effects in the cohort.

**Table 3:** SVM classification accuracies and their significance

Pairs	SVM Accuracy	*P* value	FDR-corrected *p* value	AUC	Sensitivity	Specificity
Cerebellum x Cerebellum	0.50	0.31	0.56	0.61	0.41	0.59
Basal Ganglia x Basal Ganglia	0.55	0.31	0.56	0.53	0.41	0.69
Motor Cortex x Motor Cortex	0.57	0.028*	0.085	0.55	0.34	0.79
Cerebellum x Basal Ganglia	0.69	0.0001***	0.00045***	0.73	0.59	0.79
Cerebellum x Motor Cortex	0.50	0.44	0.56	0.51	0.45	0.55
Basal Ganglia x Motor Cortex	0.45	0.70	0.70	0.47	0.45	0.45

The cerebellum includes lobules I–VI, and Crus I, and the basal ganglia includes the thalamus. FDR-correction was used as a more robust and conservative method of avoiding false positives.

*p < 0.05, ***p < 0.001.

To ensure that the classification of medication status based on BG-cerebellum features was not driven by disease severity, we conducted additional analyses to examine the correlation between clinical scores and SVM classification scores (distance to the decision boundary in the feature space) in each dataset. Our results indicate that there was no statistically significant correlation between these measures. We observed correlation coefficients of −0.25 (*p* = 0.21) for UPDRS-III scores (ON score in the medicated state), and −0.21 (*p* = 0.30), −0.21 (*p* = 0.30), 0.07 (*p* = 0.74), for tremor, bradykinesia/rigidity, and gait sub-scores, respectively. A correlation coefficient of −0.40 (*p* = 0.08) was recorded for the Montreal Cognitive Assessment (MoCA) scores^20^. These results suggest that our classification was not driven by disease severity or cognitive decline.

### Eigenvector centrality changes

**Fig. 5:**
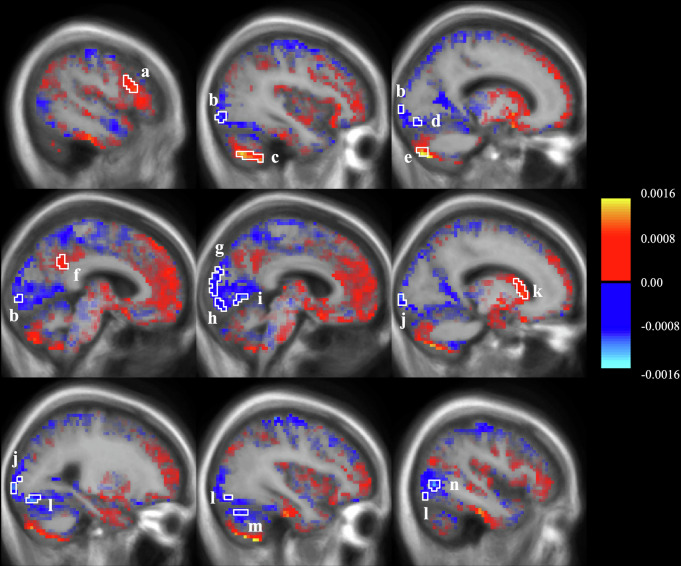
Functional connectivity changes by means of eigenvector centrality. The brain map shows the centrality score difference obtained from ON – OFF scans. Clusters reporting significant EC changes are highlighted and labelled as a–n (the letters correspond to Table 4), a significance level of *p* ≤ 0.005 (uncorrected) and 10 voxel count threshold were applied. The sagittal coordinates of the images are −51, −39, −16, −6, 6, 17, 27, 38, and 47, respectively, from left to right and top to bottom images.

To complement the seed-based analysis and identify changes in influential nodes, we used Eigenvector Centrality Mapping (ECM)^21^, a parameter-free method to measure brain connectivity patterns. We measured changes in the whole-brain EC scores from the de novo to the medicated state of the cohort at the voxel-wise level. The ECM difference between the states determines the direction of the changes. The results from one sample t-test revealed five clusters showing significant EC increases and nine clusters showing significant EC decreases at the levodopa-medicated state (Table 4). The increases were observed in the left opercular part of the inferior frontal gyrus (approximately at the Brodmann area 44; BA 44), left cerebellum, specifically lobules Crus II and VIIb, left posterior-dorsal part of the cingulate gyrus extending to the subparietal sulcus (BA 31) and right caudate nucleus (Fig. 5a, c, e, f, k, respectively). On the contrary, the decreases were found extensively in both the right and left occipital cortices, including the primary (V1), secondary (V2) and middle temporal (V5 or MT) visual areas. Additionally, right Crus I of the cerebellum also demonstrated a decrease in EC (Fig. 5m). All reported clusters showed a significant difference (*p* < 0.001 FDR-corrected at the cluster level; Table 4). We also conducted a correlation analysis to examine whether the connectivity changes of these significant clusters were associated with the changes in the MDS-UPDRS scores, but no significant correlation was observed.

**Table 4:** Summary of the significant clusters

	Cluster regions	Average ΔEC	One Sample t-Test***	Number of voxels
(a)	Left Opercular part of the inferior frontal gyrus (Brodmann area 44)	0.80 ± 0.74	*t* = 5.80	33
(b)	Left Occipital pole & Inferior occipital gyrus and sulcus (V2)	−0.89 ± 0.92	*t* = −5.22	66
(c)	Left Crus II & VIIb	1.09 ± 0.97	*t* = 5.35	25
(d)	Left Occipital pole & Inferior occipital gyrus and sulcus (V2)	−0.96 ± 1.19	*t* = −4.33	16
(e)	Left Crus II	1.26 ± 0.97	*t* = 5.80	28
(f)	Left Posterior-dorsal part of the cingulate gyrus & Subparietal sulcus(Brodmann area 31)	0.73 ± 0.89	*t* = 4.47	20
(g)	Right Cuneus (V2)	−0.85 ± 1.05	*t* = −4.38	15
(h)	Right Occipital pole & Lingual gyrus (V2)	−0.90 ± 1.09	*t* = −4.42	15
(i)	Right Lingual gyrus (V2)	−1.01 ± 1.31	*t* = −4.13	10
(j)	Right Occipital pole (V1)	−0.91 ± 1.10	*t* = −4.46	23
(k)	Right Caudate nucleus	0.92 ± 1.13	*t* = 4.37	21
(l)	Right Inferior occipital gyrus and sulcus (V2)	−0.95 ± 1.04	*t* = −4.92	41
(m)	Right Crus I	−0.83 ± 0.89	*t* = −5.04	18
(n)	Right Anterior occipital sulcus & Middle occipital gyrus (V5/MT)	−1.02 ± 1.03	*t* = −5.30	30

Average ΔEC is an average of all participants’ EC score changes at the cluster level; values are shown as mean ± standard deviation of participants’ EC score differences in ´10^3^. One sample t-test results are at the cluster level, and reported as t-statistic. All reported clusters had *p* < 0.001 (FDR-corrected). Number of voxels is the number of fMRI voxels in the cluster.

***p < 0.001.

### Correlation between changes in EC and clinical scores

**Fig. 6:**
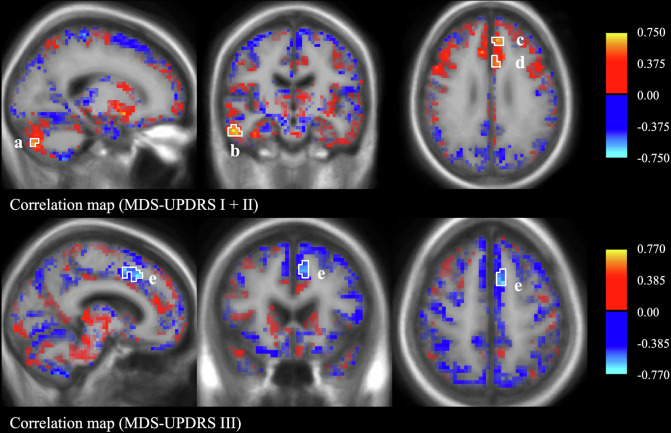
Pearson correlation between the changes in functional connectivity and MDS-UPDRS scores. The first row demonstrates the correlation between the ΔEC and ΔMDS-UPDRS I + II score. The second row shows the correlation between the ΔEC and ΔMDS-UPDRS III score. The significant clusters are highlighted and labelled as a–e (the letters correspond to Table 5), a significance level of *p* ≤ 0.005 (uncorrected) and cluster-size threshold ≥10 were applied. MNI coordinates of slice positions of the first row = (21, −17, 31); second row = (−10, 18, 50).

We further examined the association between brain connectivity and symptom severity directly through the Pearson correlation between the changes in the whole-brain voxel-wise ECM and changes in the MDS-UPDRS scores. We observed four clusters positively correlated with the combined MDS-UPDRS I + II (Fig. 6; top row). These clusters were located in the right Crus II (*r* = 0.74), right middle temporal gyrus extending to inferior temporal sulcus (BA 21; *r* = 0.73), left superior frontal gyrus to anterior cingulate gyrus and sulcus (BA 8; *r* = 0.69) and left middle-anterior cingulate gyrus and sulcus (BA 24; *r* = 0.68) (Fig. 6a–d, respectively). All of these clusters reported strong correlations with a significance level of *p* < 0.001 (FDR-corrected at the cluster level; Table 5). On the other hand, a cluster located in the left superior frontal gyrus extending to the middle-anterior cingulate gyrus and sulcus (BA 6) was negatively correlated with the MDS-UPDRS III score (*r* = −0.77, corrected *p* < 0.001; Fig. 6e; Table 5).

**Table 5:** Summary of the significant clusters

	Cluster regions	MDS-UPDRS I + II	MDS-UPDRS III	Number of voxels
(a)	Right Crus II	***r*** = 0.74***	*r* = 0.17 (*p* = 0.67)	16
(b)	Right Middle temporal gyrus & Inferior temporal sulcus (Brodmann area 21)	***r*** = 0.73***	*r* = 0.11 (*p* = 0.82)	16
(c)	Left Superior frontal gyrus & Anterior cingulate gyrus and sulcus (Brodmann area 8)	***r*** = 0.69***	*r* = −0.01 (p = 0.97)	21
(d)	Left Middle-anterior cingulate gyrus and sulcus (Brodmann area 24)	***r*** = 0.68***	*r* = 0.05 (*p* = 0.92)	14
(e)	Left Superior frontal gyrus & Middle-anterior cingulate gyrus and sulcus (Brodmann area 6)	*r* = −0.04 (*p* = 0.92)	***r*** = −0.77***	22

MDS-UPDRS I + II: correlation between the changes in the MDS-UPDRS I + II and the changes in EC of the cluster. MDS-UPDRS III: correlation between the changes in the MDS-UPDRS III and the changes in EC of the cluster; values are shown as Pearson correlation coefficient (r) and *p*-value (FDR-corrected); (***) = *p* < 0.001. Number of voxels: number of voxels in the cluster.

**Fig. 7:**
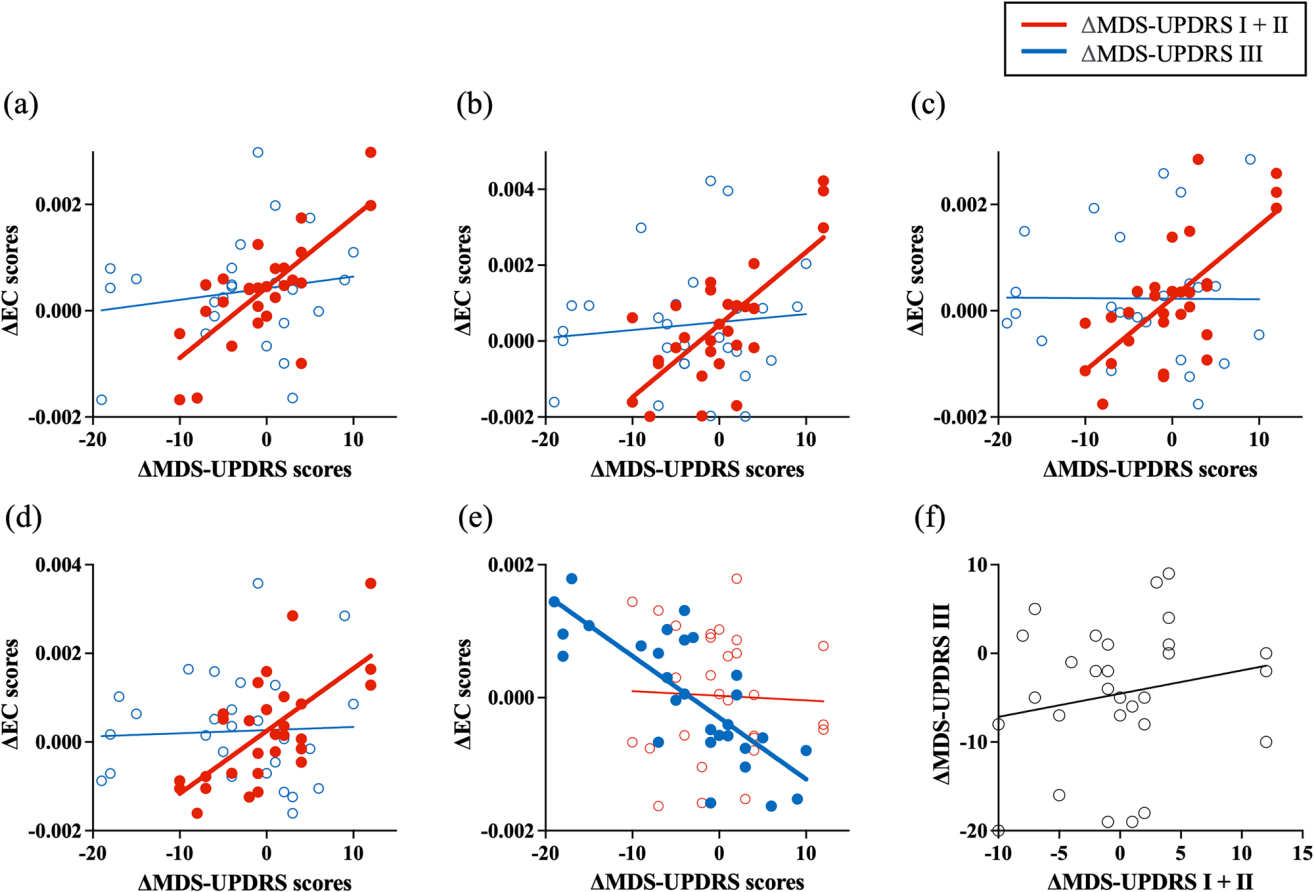
Relation between the ΔEC of the clusters and both ΔMDS-UPDRS scores. **a**–**d** The clusters demonstrate significant positive correlations with the combined MDS-UPDRS I + II. **e** The cluster shows a significant negative correlation with the MDS-UPDRS III. The clusters labelled (**a**–**e**) correspond to Fig. 6 and Table 5. **f** No correlation between the clinical outcomes. Each data point represents the score of each patient, and the line of best fit was generated by simple linear regression.

Overall, the clusters that were highly associated with the combined MDS-UPDRS I + II all showed positive correlations, meaning that as the symptoms described by the MDS-UPDRS I + II worsened, these brain regions became more functionally connected to the brain network (Fig. 7a–d). Contrarily, the left BA 6 cluster showed a negative correlation with the MDS-UPDRS III (Fig. 7e), meaning that the EC of the BA 6 reduced as motor symptom severity increased. A Pearson correlation between the changes in the MDS-UPDRS I + II and MDS-UPDRS III (Fig. 7f) suggests that there was no correlation between the combined parts I + II and the part III scores (*r* = 0.19, *p* = 0.31).

## Discussion

This study aimed to examine the long-term effect of levodopa medication in PD on neural synchrony within the motor CSTC circuitry through a longitudinal dataset. Our result suggests a significant modulatory effect on BG-cerebellum connectivity. Importantly, this effect was evident on network-level functional connectivity and not a single connection. A standard seed-based inter-regional (ROI-to-ROI) functional connectivity analysis yields no statistically significant effect of the long-term medication on either of the datasets. At the network level, the compressed feature set of BG-cerebellum connectivity was significantly different following medication in the dataset. ECM analysis revealed five functional hubs that increased connectivity in the levodopa medicated scan, of which two were in Crus II of the cerebellum and one in the caudate nucleus of the BG. However, the connectivity of multiple visual areas was weakened in that scan. Lastly, we searched for associations between changes in the connectivity patterns and changes in symptom severity. Here again, there was a strong cluster in Cerebellar Crus II showing a connectivity increase as the symptoms got worse.

The magnitude of motor response to levodopa is driven by both short-duration response (SDR) and long-duration response (LDR). While SDR is closely related to levodopa plasmatic pharmacokinetics, LDR is a sustained motor improvement induced by chronic levodopa therapy that slowly develops after treatment initiation and lasts days if discontinued^22,23^. Compared to the drug-naïve state, we found less severe overall motor symptoms at the ON-levodopa state across the group. This result is largely masked by the SDR, where the immediate symptom alleviation roughly parallels the plasma levodopa level. Yet, there is notion that the symptomatic effects of levodopa may delay the natural progression of motor disability through complex mechanisms of LDR^24^. Cilia et al.^24^ showed a 31% lower annual decline of UPDRS-III scores in the OFF-levodopa state over a 24-month treatment duration relative to the natural progression (consecutive drug-naïve) of motor symptoms.

The SVM-based classification analysis pointed to the relevance of cerebellum-BG network connectivity in discriminating PD medication status (de novo versus levodopa-medicated). The interaction between the cerebellum and BG has traditionally been thought to occur at the cortical level, as both areas form loops with the cortex (for review see Haar and Donchin^25^). However, recent anatomical tracing studies have demonstrated bidirectional pathways facilitating a direct cerebellum-BG interplay, which could lead to the formation of an integrated functional network^18,26,27^. Accordingly, resting state striatum-cerebellum functional coupling has been indicated in healthy adults, where the connections are segregated based on functional topography and decline in advanced age, where dopamine has a normative decline^28,29^. Further, levodopa was shown to enhance the connectivity in motor pathways joining putamen with the cerebellum and brainstem in healthy individuals^30^. These findings led to the speculation that dopamine may be particularly important in modulating the functional interaction between the cerebellum and BG.

In PD, heterogenous results were reported. A recent study by Bagarinao et al.^14^ demonstrated significantly impaired connectivity between cerebellar connector hubs and BG in PD, with altered connectivity correlating with clinical manifestations. Hacker et al.^4^ found markedly lower connectivity between the cerebellum with caudate and putamen in advanced, medicated patients relative to matched controls. In contrast, Helmich et al.^9^ found no difference in striatum-cerebellum connectivity between de novo or off-medication patients and healthy controls. Gao et al.^31^ reported decreased cerebellar connectivity with putamen and GP in off-levodopa patients and subsequent normalisation following levodopa administration. Simioni et al.^32^ described a contrasting pattern of increased coherence between putamen and motor cerebellum in the off- medication mild-moderate patients and the normalising effect of levodopa. The inconsistent results could be due to the differential role of cerebellum-BG interaction at different disease stages and the variations in ROI choice and localisation. It is also possible that the variations originated from a focus on individual connections, and a potentially more coherent network modulation of levodopa might have been missed.

In our study, while pure univariate effects were absent from interregional cerebellum-BG connectivity in differentiating de novo and levodopa conditions, the multivariate combination of individual connections demonstrated robust discriminative power. This indicates a contrast in a distributed pattern of neural synchrony between the medication conditions. Specifically, a network of intra- and cross-hemispheric connectivity between cerebellar and BG nodes acted synergistically to manifest a difference that was not accessible to univariate comparisons. Complementing the previous work investigating connectivity between specific brain regions^29,30^, our finding provides new insight into dopamine’s potentially wider, system-level modulation of the reciprocally connected cerebellum-BG network. In PD, increased striatal dopamine following levodopa medication could prevent transmitting aberrant BG signals to the cerebellum that could evoke cerebellar hyperactivity and disrupt cerebello-thalamo-cortical pathways^17,18,33^. A refined cerebellum-BG interaction could also facilitate the combination of supervised learning and reinforcement learning (specialised by cerebellum and BG, respectively) and consequently lead to better motor learning and adaptation in PD^18^.

Finally, the levodopa-induced modification of cerebellar-BG connectivity found here concurs with previous works that found a similar effect on this pathway following effective Deep Brain Stimulation (DBS) therapy in PD. Kahan et al.^34^ conducted dynamic causal modelling in DBS patients and showed that only during a motor task (but not during rest) did the DBS modulate the cerebellum-BG connectivity. A more recent investigation of STN DBS in PD highlighted the importance of cerebellum and cerebellum-BG interaction in PD symptom alleviation^35^. This study found that DBS modulate the functional architecture of large-scale brain networks, including the restoration of lowered dynamic FC between the cerebellum and BG and motor thalamus^35^.

While none of the changes on the ROI-to-ROI levels survived the correction for multiple comparisons, we observed a few trends that are likely to contribute to the significant network effect and present some interesting links to previous literature.

First, de novo patients were inclined to exhibit higher connectivity between left cerebellar lobules I–IV and the motor cortex. Cerebellar lobules I–IV are considered to be primarily involved in motor processing and cerebellar hyperactivations in PD were consistently reported during the execution of various upper limb movements and motor learning^36–38^. In a PET study, early PD patients showed additional activations of the bilateral cerebellum while achieving equal performance in motor sequence learning as the control participants^39^. In agreement with our results, Wu et al.^40^ showed that levodopa normalises the heightened rs-FC in the cerebellum and primary motor cortex in PD. The physiological significance of strengthened connectivity and hyperactivity of the cerebellum might be interpreted as compensatory mechanisms in response to impaired subcortical-cortical loops. It was suggested that by restoring the motor circuits with levodopa, patients might become less reliant on the compensatory effects^40^.

Second, we observed a trend of attenuated rs-FC among the thalamus, putamen, and GP at the second time point. Contrary to our observation, previous studies have shown an acute levodopa effect on improving the deficient rs-FC among BG and thalamus nodes^41^. In a longitudinal investigation, Li et al. reported a time-related decline in BG connectivity focused on putamen in PD, which was associated with changes in nigrostriatal dopaminergic integrity^42^. Hence, our results could represent the net effect of medication and progressing dopaminergic dysfunction that cannot be effectively disentangled.

Third, the patients tend to exhibit elevated connectivity between GP and motor areas, including supplementary motor area (SMA), premotor, and primary motor cortices. A leading hypothesis of PD pathophysiology is the imbalance between direct and indirect BG pathways due to DA deficiency. Suppression of GP interna (GPi; BG output nucleus) and stimulation of GP externa (GPe) firings in PD were shown to alleviate the motor symptoms, which was presumably due to selective inhibition of the indirect pathway^43,44^. Studies in pallidotomy and GPi deep brain stimulation (DBS) patients have shown that metabolic activities in frontal motor areas were reinstated after the procedures, indicating the excessive pallidal inhibition of the thalamocortical system in PD^45–47^. Considering the above, our findings might reflect a modulatory effect of levodopa on BG pathways, resulting in enhanced GPe-motor cortex connectivity and attenuated GPi-cortex anti-coherence. Nevertheless, a clear inference cannot be drawn as we did not differentiate GPi and GPe despite their distinctive roles in the BG circuitry.

Fourth, the patients had a trend of decreasing intra-cerebellar rs-FC, particularly within and between lobules VI and Crus I. This is consistent with the study by Festini et al., wherein an increase in the connectivity between lobule VI and Crus I and neighbouring regions was found in patients OFF medication compared to ON^48^. Additionally, they found no intra-cerebellar connections were stronger at ON compared to OFF. However, it is important to note that in the Festini et al. the ON versus OFF contrast was conducted within the same day, where “OFF” was a temporary withdrawal rather than drug naïve. In contrast, our study design reflects the long-term effects of medication. This suggests unlike in the BG (the second point above), in the cerebellum, SDR and LDR changes in connectivity are similar.

Using ECM to study the voxel-wise level connectivity pattern, we observed five clusters presenting an increase and nine presenting a decrease in connectivity at the levodopa state compared to the drug naïve state. Two significant clusters were found in the left cerebellum, at lobules VIIb and Crus II, and presented the largest EC increase. Previous studies showed a connectivity increase following an acute levodopa challenge in many cerebellar lobules which play a role in motor function^7,49,50^, including lobule VIIb which is associated with lower-limb movement^51,52^.

Another cerebellar cluster was in the right Crus I, but it showed decreased EC following medication. Interestingly, research on the PPMI data also showed a significant decline in the Crus I anatomical volume as a result of PD progression^13^. Cerebellar Crus I is considered to be related to cognitive domains^52^, including executive function, working memory, language processing, visuospatial function, and attention^53,54^. Accordingly, this FC decrease might be due to visual disturbance as a result of disease progression which has been shown to relate to dementia in PD^55–57^.

The ECM analysis also found a connectivity increase in the right caudate nucleus at the medicated state. In line with our network findings of the modulatory effect of levodopa on the FC of the cerebellum and BG.

While our ROIs and network analysis focused on the motor CSTC circuitry, our voxel-wise ECM analysis revealed multiple additional cortical nodes. Increased EC was evident in the left dorsal posterior cingulate gyrus and the left opercular part of the inferior frontal gyrus, which previously showed increased activity in PD patients^58^. On the other hand, we found a large connectivity decrease at the levodopa state across several occipital regions, affecting most of the visual areas including V1, V2, and V5 on both the left and right hemispheres. Weakened FC in occipital regions in the OFF-levodopa PD patients compared to healthy individuals, was previously observed by Zhong et al.^50^ using distant degree centrality analysis. The FC decrease in the visual cortices in PD has also been evidenced by several studies using different FC analysis approaches^50,56,59^.

Considering the variability across the cohort in the amount and direction of the changes in symptoms between the two scans, we further looked at the correlations between the changes in clinical outcomes and the changes in EC. Here we found four clusters showing a positive correlation with UPDRS I + II – which captures motor and non-motor experiences of daily living and was recently selected as an outcome measure for upcoming clinical trials in PD – and a single cluster showing a negative correlation with UPDRS III – which captures motor symptoms assessment.

The single cluster correlating with the motor symptoms assessment was detected in the medial portion of the left BA 6, in between the superior frontal and middle-anterior cingulate gyri, which is known as the SMA. It has been apparent that the motor cortices are functionally altered in PD, including the primary motor cortex, sensorimotor cortex and supplementary motor cortex^60–62^. Dysfunction of the SMA was found to correlate with gait impairment, disruption of temporal processing, and motor sequencing deficit in PD^63^. The result demonstrated a negative correlation between the SMA connectivity and the MDS-UPDRS III score, suggesting that the motor symptom severity of PD patients reduced when the FC of the SMA increased.

Contrarily, the clusters positively correlated with the MDS-UPDRS I + II score were mostly detected in regions involved in cognitive function. These regions include the middle temporal gyrus (BA 21) which plays a role in visual perception^64^, the frontal eye field in the BA 8^65^ and the BA 24 which is involved in goal-directed behaviours^66^. Cerebellar right Crus II also demonstrated a positive correlation with the MDS-UPDRS I + II score. Altogether, we can infer that the connectivity of these cognitive areas increased as the non-motor symptoms captured by the MDS-UPDRS I + II worsened.

This study has a couple of limitations. First, the causal influence of levodopa on clinical and connectivity characteristics cannot be established with an observational design. Second, no ROI-to-ROI rs-FC change survived FDR correction for multiple comparisons to prevent the occurrence of type I errors. This could be due to the inadequate sample size and, therefore, a lower statistical power. Third, given that levodopa patients were ON medication during fMRI scans, our results reflected a mixture of short- and long-duration medication effects on functional reorganisation in the brain that cannot be disentangled. Finally, the field of view of the fMRI scan had incomplete coverage of the posterior cerebellum in some patients. This issue was addressed by including only the lobules above the horizontal fissure in the ROI analysis and setting a threshold for voxel inclusion for the voxel-wise ECM analysis (Supplementary Fig. 1).

To conclude, we demonstrated the modulatory effect of levodopa on rs-FC, which is strongest between the cerebellum and BG networks. This effect was significant only in the network analysis indicating that levodopa modulated collective patterns of BG-cerebellum neural synchrony. Following the recent evidence suggesting the bidirectional linkage between the cerebellum and BG networks, our results provide further insight into the relevance of the inter-network functional connectivity in Parkinson’s, as well as in the brain functional reorganisation processes. Furthermore, our ECM analysis demonstrated that levodopa strengthened the connectivity of functional hubs in the cerebellum and the BG, and increased connectivity in the SMA was associated with improved motor symptoms. On the contrary, a significant connectivity decrease between the scans was observed in brain regions involved in visual processing and certain brain regions involved in cognitive function demonstrated correlations between an increase in connectivity to worsened experiences of daily living.

## Methods

### Participants

The clinical and rs-fMRI data were extracted from the Parkinson’s Progression Marker Initiative (PPMI; see https://www.ppmi-info.org/), a muti-centre observational study of clinical and neuroimaging progression markers of PD conducted since 2010. The study design and protocol are available online (https://www.ppmi-info.org/study-design). The study and the data collection have been performed in accordance with the Declaration of Helsinki and were approved by the institutional review board or local ethics committee at each of the participating sites (https://www.ppmi-info.org/about-ppmi/ppmi-clinical-sites). All PPMI participants provided written informed consent. The PPMI study recruited PD participants who (1) had at least two of the cardinal motor symptoms (resting tremor, bradykinesia, and rigidity); (2) had a Hoehn and Yahr progression score of 1 or 2; (3) were not expected to initiate PD medication within 6 months from baseline; (4) were diagnosed within 2 years before the entry and aged at least 30 years at the time of diagnoses. Patients were excluded if they (1) had taken levodopa, dopamine agonists, monoamine oxidase-B inhibitors, or amantadine within 60 days to baseline; and (2) have taken levodopa or dopamine agonists for longer than 60 days prior to baseline.

At the time we downloaded the data, a total of 135 patients had had at least one rs-fMRI scan. Here, we extracted a longitudinal group where the patients had had at least two rs-fMRI scans, first during the drug-naïve phase and second after transitioning into the levodopa-medicated state (co-administered with a dopa-decarboxylase inhibitor; not on other PD medications), with a moderately concentrated distribution of treatment durations at the second visit across the samples (*N* = 29).

### Clinical measurements

All participants underwent motor and neuropsychological examinations at the time of selected visits. The presence of motor signs was evaluated using Movement Disorder Society-Unified PD Rating Scale (MDS-UPDRS)^19^. We subdivided the UPDRS scale into a tremor score (items 2.10 and 3.15–3.18), a bradykinesia and rigidity score (items 3.3-3.8), as well as a gait disturbance score (items 2.12, 2.13, 3.10, 3.11, and 3.12) based on previous studies^67,68^. For the non-motor dimension, we included the Montreal Cognitive Assessment (MoCA)^20^. We further extracted the sum score of the MDS-UPDRS parts I + II and the total MDS-UPDRS part III score for correlation analysis of the selected cohort. The total MDS-UPDRS III score is the common measure for motor symptoms, based on assessment by a clinician. The combined MDS-UPDRS I + II score represented long-term PD progression, reflecting both motor and non-motor symptoms, as reported in Gonzalez-Robles et al.^69^.

### Imaging acquisition, pre-processing, and region-of-interest selection

Whole-brain T1-weighted anatomical MRI and rs-fMRI scans using Siemens Trio Tim 3 Tesla magnets (Siemens Medical Solutions, Erlangen, Germany) were acquired from the PPMI database. Imaging parameters were identical across the clinical sites. Briefly, T1-weighted high-resolution anatomical image (voxel size: 1 × 1 × 1 mm^3^) was obtained for each patient with repetition time (TR) = 2300 ms, echo time (TE) = 2.98 ms, flip angle (FA) = 9°. rs-fMRI echo-planer scans were conducted in 8.5 min with 210 time points, TR = 2400 ms, TE = 25 ms, FA = 80°, voxel size = 3.25 × 3.25 × 3.25 mm^3^.

Anatomical reconstruction (cortical) and segmentation (subcortical) were performed using FreeSurfer (version 7.4.1; https://surfer.nmr.mgh.harvard.edu/). The processes included motion correction, removal of non-brain tissues, intensity normalisation, and classification of voxels into white matter (WM) and grey matter (GM) based on intensity and neighbour constraints. In the surface-based stream, the WM and pial surfaces were constructed and refined to follow the intensity gradients between WM and GM and between GM and cerebrospinal fluid, respectively^70^. Individual surfaces were then aligned to the spherical Destrieux atlas through a non-linear registration algorithm, which fitted cortical folding patterns to an average cortical geometry with each hemispheric cortex parcellated into 74 regions^71,72^. In the volume-based stream, the subcortical regions were segmented and labelled in the native space^73,74^. FreeSurfer longitudinal pipeline was used to construct an unbiased participant-specific template through an inverse consistent registration between the two scans^75^. The common information on within-participant templates then initialised the image processing steps (see above) for scans at each visit^76^.

Rs-fMRI pre-processing was conducted using FreeSurfer FS-FAST (https://surfer.nmr.mgh.harvard.edu/fswiki/FsFastTutorialV6.0/FsFastPreProc/). The procedure entailed normalising intensity, motion correction, and slice timing correction. Pre-processed anatomical volumes in native space were resampled into native rs-fMRI space using mri_vol2vol and bbregister to extract the corresponding time courses of segmented regions. Pre-processed volumes were also resampled to the MNI305 space.

We defined subcortical and cortical ROIs comprising the motor CSTC circuitry, along with cerebellar ROIs (Table 2). The motor ROI cluster included bilateral precentral gyri (PreCG), inferior precentral sulci (InfPreCS), superior precentral sulci (SupPreCS), caudal superior frontal gyri (SFGcau; posterior 1/3 of superior frontal gyrus), and caudal middle frontal gyri (MFGcau; posterior 1/3 of middle frontal gyrus) based on the Destrieux atlas parcellation^72^. PreCG, InfPreCS, and SupPreCS were localised in the main body, anteroinferior, and anterosuperior subsections of the primary motor cortex, respectively. SFGcau corresponded to the anatomical location of the SMA, and MFGcau was functionally mapped to premotor cortices. At the subcortical level, we focused on the bilateral thalamus, caudate nucleus, putamen, globus pallidus, and cerebellar cortex.

For the cerebellar ROIs, SUIT atlas of the cerebellum^77^ was overlayed on the preprocessed rs-fMRI data in the MNI305 space. A threshold of 70% was set for the cerebellar coverage for every rs-fMRI used in the analysis, which resulted in lobules above the horizontal fissure being included in the study. These lobules are I–IV, V, I, and Crus I for the left and right hemispheres.

### Statistical analysis

#### First-level analysis of rs-fMRI data

All analyses were conducted using MATLAB R2021a (https://uk.mathworks.com/products/matlab.html). Voxel- (volume space) and vertex-level (surface space) time-series data for each ROI, cerebrospinal fluid (CSF), and WM were extracted, along with the whole-brain average time course. The first four images of the fMRI time-series were discarded. To exclude spurious signal confounds, we implemented a stepwise noise attenuation procedure. First, a high-pass filter with a threshold of 0.01 Hz was used, and the six head motion parameters estimated by FreeSurfer were regressed out of all raw fMRI data through linear regression. The voxel-level time courses were averaged for CSF and WM, then regressed out from all voxels and vertices that constitute each ROI. Next, the cleaned residuals from the fits were averaged to create ROI time traces. Lastly, before mean-centring and standardisation, we further denoised the ROI time series with a low-pass filter retaining frequencies below 0.20 Hz to remove the potential high-frequency fluctuations injected in partialling-out processes. The pairwise Pearson correlation coefficients between the filtered time-series of each ROI were computed and stored in a 26 × 26 symmetric FC matrix based on the selected ROI mentioned in paragraph 5.3.3.

#### Second level within- and between-group comparison

Nonparametric permutation testing was used to extract significant differences in the connectivity maps of patients in de novo and levodopa-medicated states. The permutation inferences assume that data can be arbitrarily exchanged without affecting the joint probability distribution^78^. Nevertheless, given the presence of nuisance covariates, such as disease duration, age, and gender, the data cannot be considered exchangeable even under the null hypothesis. In this respect, the Freedman-Lane procedure was followed to compute the estimates of null distribution to ascribe *p*-values for each rs-FC^78–80^. First, we fitted a full general linear model (GLM):

1 $$Y=X\beta +N\gamma +\varepsilon$$

The $Y$ included the within-participant contrast of each connectivity (levodopa $\mbox{--}$ de novo), $X$ was a vector of ones (the overall mean effect), and $N$ contained the difference in disease duration between two timepoints, baseline age, and gender. Parameters $\beta$ and $\gamma$ were, respectively, for factors of interest and nuisance variables, and $\varepsilon$ represented the residual. We then created a reduced GLM by regressing $Y$ only with nuisance variables and obtained estimated regression parameters ${\hat{\gamma }}_{N}$ and nuisance-only residuals ${\hat{\varepsilon }}_{N}$ [variance of the null model (Eq. 2)]. Here, the mean connectivity difference ${\bar{Y}}_{0}$ was derived as test-statistics ${T}_{0}$.

2 $$Y=N{\hat{\gamma }}_{N}+{\hat{\varepsilon }}_{N}$$

Next, we shuffled the residual vector by multiplying by a permutation matrix ${P}_{j}$. The nuisance signal estimated in the earlier step, $N{\hat{\gamma }}_{N}$, was then added back to the permuted residuals, ${{P}_{j}\hat{\varepsilon }}_{N}$, to produce the permuted estimations of connectivity contrasts ${Y}_{j}$:

3 $${Y}_{j}=N{\hat{\gamma }}_{N}+{{P}_{j}\hat{\varepsilon }}_{N}$$

Finally, the permuted estimates were regressed against the full model (Eq. 4), including both primary and nuisance factors, and the test-statistics ${T}_{j}$ were computed.

4 $${Y}_{j}=X\beta +N\gamma +\varepsilon$$

The permutation was repeated 10,000 times to generate the null distribution of test statistics. Each FC was deemed significantly different (uncorrected) between the medication states if the absolute observed ${T}_{0}$ was greater than 95% of the absolute values of distributed ${T}_{j}$. Finally, the FDR-based multiple comparison procedure was adopted to adjust the derived *p*-values^81^.

#### Network level testing of medication effect on functional connectivity

To evaluate the discriminating power of neural synchrony at the network level, we consider three subnetworks of the motor CSTC circuitry: cortical motor areas, sub-cortical motor areas (BG and thalamus), and cerebellum. For all three subnetworks, we integrated the hemispheres. We then constructed SVM implemented on within- and cross-network features to classify patients with respect to medication status. We first took all pairwise combinations of the networks as masks, and participant connectivity values in each mask were collapsed into a feature vector. To this end, five feature vectors (one for each of the within and between networks connections) with varying lengths (according to the number of ROIs in each network) were retrieved, with the numbers of FC values in most vectors outnumbering the number of observations.

#### Dimensionality reduction

To attenuate the risk of overfitting and ensure the predictive algorithms based on different network-level feature sets are comparable, we used principal component analysis to linearly transform correlated FC values into a reduced number of orthogonal variables, i.e., principal components (PC), to derive new vector sets with the same dimensionality^82^. In specific, eigen decomposition of the covariance matrices from each standardised feature set was performed to compute eigenvalues and eigenvectors. The eigenvalues were then ranked in a descending order effectively representing decreasing variance in the data carried by the corresponding PCs, whose directions were represented by the associated eigenvectors^82,83^. For within subnetwork of FC values, all the features were used; whereas for the between subnetworks, the number of PCs was *n-1* where *n* is the number of features.

#### Support vector machine and cross-validation

Binary classifications of de novo and levodopa-medicated patients were performed using SVMs with the linear kernel through the *fitcsvm* route in MATLAB. Detailed documentation of SVM and the optimisation problem can be found in Cortes et al.^84^. Briefly, a linear SVM projects the training data points into a high-dimensional feature space and seeks an optimal hyperplane with the maximal margin separating the two classes^84^. The optimal hyperplane is defined as:

5 $$f\left(x\right)={w}^{T}{x}_{i}+b=0$$

where $w$ is the weight vector perpendicular to the hyperplane, $b$ is the bias term, ${x}_{i}$ is $i$-th input vector in the dataset, and $f(x)$ is the linear discriminant function whose sign represents the class of training inputs^84^. The objective of maximising the geometric margin in the feature space corresponds to the primal optimisation problem (which is then reformulated into the Lagrangian dual function), $\mathop{\min }\limits_{w}\frac{1}{2}{w}^{T}w$, subject to,$\,{y}_{i}\left({w}^{T}{x}_{i}+b\right)\ge 1$.

The SVM models were evaluated with leave-one-out cross-validation (LOOCV). A subsample contained data of the same patient measured at two medication conditions. Permutation testing was applied to assess whether each classifier captured a real class structure in the data. Here, we permuted the class labels and refitted each SVM 10,000 times to estimate the corresponding null distribution of accuracy values. The accuracy was considered significantly higher than chance if it exceeded the 97.5th percentile of the null distribution. For the classifiers with above-chance accuracy, we computed the area under the receiver operating characteristic curve (AUC) that aggregates the classifying efficacy under all possible decision thresholds.

#### Eigenvector centrality mapping

For ECM analysis, the fMRI data in the MNI305 space was spatially smoothed with 5 mm full-width half maximum, and resampled to a 3 × 3 × 3 mm^3^ isotropic voxel grid. For time series pre-processing, we first performed motion regression using six motion parameters obtained from the FS-FAST processing. Then, the average white matter (WM) and cerebrospinal fluid (CSF) signals were regressed out of each voxel time series as nuisance variables. A band-pass filter [0.01–0.2 Hz] was then applied, and finally, the data was scaled using Z-score normalisation.

A total of approximately 30,000 voxels were extracted per scan covering the entire brain, including the subcortical, cortical and cerebellar regions, but excluding the WM. Pearson correlation was used to compute a similarity matrix $A=n\times n$, where $n\approx$ 30,000 nodes. The pairwise correlation coefficients between voxel time series represented the weights along the graph edges, typically holding non-negative values^21^. Thus, we took absolute values of the correlation coefficients for EC computation to retain only the positive weights. Lastly, EC mapping was achieved using the Brain Connectivity Toolbox in MATLAB^85^.

To identify brain connectivity changes from de-novo to levodopa medicated state, we first calculated the voxel-wise differences of EC, where $\Delta EC=E{C}_{Ldopa}-E{C}_{de-novo}$, generating an EC difference map for each participant. To handle missing fMRI data in the cerebellar regions due to limited fMRI acquisition, a threshold of 15 participants per voxel was applied to include most cerebellar voxels in the analysis. Meaning, each voxel must be present in the scans of most participants (at least 15) to be included in subsequent analysis (see Supplementary Fig. 1). Voxel-wise one sample t-test was then conducted to test whether the EC difference of each voxel was significantly different from zero. A significance level of *p* ≤ 0.005 (uncorrected) and a cluster-size threshold of >10 voxels were used to define significant clusters. FDR method was further used to correct for multiple comparisons at the cluster level with a *p* ≤ 0.005 significance level.

We further investigated the correlation between the whole-brain voxel-wise ECM changes and clinical outcomes. Pearson correlation coefficients were computed between the voxel-wise EC difference maps of all participants to the $\Delta$MDS-UPDRS I + II and $\Delta$MDS-UPDRS III separately, generating two correlation maps. The significant clusters were defined using a *p* ≤ 0.005 (uncorrected) and a cluster-size threshold of 10 voxels. Finally, FDR correction was applied at the cluster level.

## Supplementary information

Supplementary Figure 1

## Data Availability

The datasets analysed during the current study are fully available in the Parkinson’s Progression Markers Initiative (PPMI) database (https://www.ppmi-info.org/).

## Code availability

The underlying code for this study will be made available to qualified researchers at reasonable request from the corresponding author.

## References

[1] Shastry, B. S. Parkinson disease: etiology, pathogenesis and future of gene therapy. *Neurosci. Res.* **41**, 5–12 (2001). 11535288 10.1016/s0168-0102(01)00254-1

[2] Haddad, F., Sawalha, M., Khawaja, Y., Najjar, A. & Karaman, R. Dopamine and levodopa prodrugs for the treatment of Parkinson’s disease. *Molecules* **23**, 40 (2017). 29295587 10.3390/molecules23010040 PMC5943940

[3] Braak, H., Ghebremedhin, E., Rüb, U., Bratzke, H. & Del Tredici, K. Stages in the development of Parkinson’s disease-related pathology. *Cell Tissue Res.* **318**, 121–134 (2004). 15338272 10.1007/s00441-004-0956-9

[4] Hacker, C. D., Perlmutter, J. S., Criswell, S. R., Ances, B. M. & Snyder, A. Z. Resting state functional connectivity of the striatum in Parkinson’s disease. *Brain* **135**, 3699–3711 (2012). 23195207 10.1093/brain/aws281 PMC3525055

[5] Poewe, W. & Espay, A. J. Long duration response in Parkinson’s disease: levodopa revisited. *Brain* **143**, 2332–2335 (2020). 32844192 10.1093/brain/awaa226

[6] Thanvi, B. R. & Lo, T. C. N. Long term motor complications of levodopa: clinical features, mechanisms, and management strategies. *Postgrad. Med. J.* **80**, 452–458 (2004). 15299154 10.1136/pgmj.2003.013912 PMC1743071

[7] Mueller, K. et al. Modulatory effects of levodopa on cerebellar connectivity in Parkinson’s disease. *Cerebellum* **18**, 212–224 (2019). 30298443 10.1007/s12311-018-0981-y PMC6443641

[8] Warren, J. D. et al. Molecular nexopathies: a new paradigm of neurodegenerative disease. *Trends Neurosci.* **36**, 561–569 (2013). 23876425 10.1016/j.tins.2013.06.007 PMC3794159

[9] Helmich, R. C. et al. Spatial remapping of cortico-striatal connectivity in Parkinson’s disease. *Cereb. Cortex* **20**, 1175–1186 (2010). 19710357 10.1093/cercor/bhp178

[10] Luo, C. et al. Reduced functional connectivity in early-stage drug-naive Parkinson’s disease: a resting-state fMRI study. *Neurobiol. Aging* **35**, 431–441 (2014). 24074808 10.1016/j.neurobiolaging.2013.08.018

[11] Tahmasian, M. et al. A systematic review on the applications of resting-state fMRI in Parkinson’s disease: does dopamine replacement therapy play a role?. *Cortex* **73**, 80–105 (2015). 26386442 10.1016/j.cortex.2015.08.005

[12] Wu, T. & Hallett, M. The cerebellum in Parkinson’s disease. *Brain* **136**, 696–709 (2013). 23404337 10.1093/brain/aws360 PMC7273201

[13] Yun, J. J., Taurines, A. G. de, Tai, Y. F. & Haar, S. Anatomical abnormalities suggest a compensatory role of the cerebellum in early Parkinson’s disease. *NeuroImage*, **310**, 121121 (2024). 10.1016/j.neuroimage.2025.121121 40054760

[14] Bagarinao, E. et al. Connectivity impairment of cerebellar and sensorimotor connector hubs in Parkinson’s disease. *Brain Commun.* **4**, fcac214 (2022). 36072644 10.1093/braincomms/fcac214 PMC9438962

[15] Jahanshahi, M. et al. Dopaminergic modulation of striato-frontal connectivity during motor timing in Parkinson’s disease. *Brain* **133**, 727–745 (2010). 20305278 10.1093/brain/awq012

[16] Wu, T. et al. Effective connectivity of brain networks during self-initiated movement in Parkinson’s disease. *NeuroImage* **55**, 204–215 (2011). 21126588 10.1016/j.neuroimage.2010.11.074

[17] Bostan, A. C. & Strick, P. L. The cerebellum and basal ganglia are interconnected. *Neuropsychol. Rev.* **20**, 261–270 (2010). 20811947 10.1007/s11065-010-9143-9 PMC3325093

[18] Caligiore, D. et al. Consensus paper: towards a systems-level view of cerebellar function: the interplay between cerebellum, basal ganglia, and cortex. *Cerebellum* **16**, 203–229 (2017). 26873754 10.1007/s12311-016-0763-3 PMC5243918

[19] Goetz, C. G. et al. Movement Disorder Society-sponsored revision of the Unified Parkinson’s Disease Rating Scale (MDS-UPDRS): scale presentation and clinimetric testing results. *Mov. Disord.* **23**, 2129–2170 (2008). 19025984 10.1002/mds.22340

[20] Nasreddine, Z. S. et al. The Montreal Cognitive Assessment, MoCA: a brief screening tool for mild cognitive impairment. *J. Am. Geriatr. Soc.* **53**, 695–699 (2005). 15817019 10.1111/j.1532-5415.2005.53221.x

[21] Lohmann, G. et al. Eigenvector centrality mapping for analyzing connectivity patterns in fMRI data of the human brain. *PLoS ONE* **5**, e10232 (2010). 20436911 10.1371/journal.pone.0010232 PMC2860504

[22] Nutt, J. G. & Holford, N. H. The response to levodopa in Parkinson’s disease: imposing pharmacological law and order. *Ann. Neurol.* **39**, 561–573 (1996). 8619540 10.1002/ana.410390504

[23] Wider, C. et al. Long-duration response to levodopa in patients with advanced Parkinson disease treated with subthalamic deep brain stimulation. *Arch. Neurol.* **63**, 951–955 (2006). 16831963 10.1001/archneur.63.7.951

[24] Cilia, R. et al. Natural history of motor symptoms in Parkinson’s disease and the long-duration response to levodopa. *Brain* **143**, 2490–2501 (2020). 32844196 10.1093/brain/awaa181 PMC7566883

[25] Haar, S. & Donchin, O. A revised computational neuroanatomy for motor control. *J. Cognit. Neurosci.* **32**, 1823–1836 (2020). 32644882 10.1162/jocn_a_01602

[26] Hoshi, E., Tremblay, L., Féger, J., Carras, P. L. & Strick, P. L. The cerebellum communicates with the basal ganglia. *Nat. Neurosci.* **8**, 1491–1493 (2005). 10.1038/nn1544 16205719

[27] Pelzer, C. et al. The protease activity of the paracaspase MALT1 is controlled by monoubiquitination. *Nat. Immunol.* **14**, 337–345 (2013). 23416615 10.1038/ni.2540

[28] Bernard, J. A. et al. Resting state cortico-cerebellar functional connectivity networks: a comparison of anatomical and self-organizing map approaches. *Front. Neuroanat*. **6**, 31 (2012). 10.3389/fnana.2012.00031 PMC3415673 22907994

[29] Hausman, H. K., Jackson, T. B., Goen, J. R. M. & Bernard, J. A. From synchrony to asynchrony: cerebellar-basal ganglia functional circuits in young and older adults. *Cereb. Cortex* **30**, 718–729 (2020). 31219563 10.1093/cercor/bhz121

[30] Kelly, C. et al. L-dopa modulates functional connectivity in striatal cognitive and motor networks: a double-blind placebo-controlled study. *J. Neurosci.* **29**, 7364–7378 (2009). 19494158 10.1523/JNEUROSCI.0810-09.2009 PMC2928147

[31] Gao, L.-L., Zhang, J.-R., Chan, P. & Wu, T. Levodopa effect on basal ganglia motor circuit in Parkinson’s disease. *CNS Neurosci. Ther.* **23**, 76–86 (2017). 27663605 10.1111/cns.12634 PMC6492721

[32] Simioni, A. C., Dagher, A. & Fellows, L. K. Compensatory striatal–cerebellar connectivity in mild–moderate Parkinson’s disease. *NeuroImage: Clin.* **10**, 54–62 (2016). 26702396 10.1016/j.nicl.2015.11.005 PMC4669533

[33] Milardi, D. et al. The cortico-basal ganglia-cerebellar network: past, present and future perspectives. *Front Syst. Neurosci.* **13**, 61 (2019). 31736719 10.3389/fnsys.2019.00061 PMC6831548

[34] Kahan, J. et al. Deep brain stimulation has state-dependent effects on motor connectivity in Parkinson’s disease. *Brain* **142**, 2417–2431 (2019). 31219504 10.1093/brain/awz164 PMC7053573

[35] Chu, C. et al. Subthalamic stimulation modulates motor network in Parkinson’s disease: recover, relieve and remodel. *Brain* **146**, 2780–2791 (2023). 36623929 10.1093/brain/awad004

[36] Guell, X. & Schmahmann, J. D. fMRI-based anatomy: mapping the cerebellum. in *Essentials of Cerebellum and Cerebellar Disorders* 351–356. 10.1007/978-3-031-15070-8_54 (Springer, 2023).

[37] Rascol, O. et al. The ipsilateral cerebellar hemisphere is overactive during hand movements in akinetic parkinsonian patients. *Brain* **120**, 103–110 (1997). 9055801 10.1093/brain/120.1.103

[38] Wu, T., Wang, L., Hallett, M., Li, K. & Chan, P. Neural correlates of bimanual anti-phase and in-phase movements in Parkinson’s disease. *Brain A J. Neurol.* **133**, 2394–2409 (2010). 10.1093/brain/awq151 PMC3139934 20566485

[39] Mentis, M. J. et al. Early stage Parkinson’s disease patients and normal volunteers: comparative mechanisms of sequence learning. *Hum. Brain Mapp.* **20**, 246–258 (2003). 14673808 10.1002/hbm.10142 PMC6871797

[40] Wu, T. et al. Changes of functional connectivity of the motor network in the resting state in Parkinson’s disease. *Neurosci. Lett.* **460**, 6–10 (2009). 19463891 10.1016/j.neulet.2009.05.046

[41] Szewczyk-Krolikowski, K. et al. Functional connectivity in the basal ganglia network differentiates PD patients from controls. *Neurology* **83**, 208 (2014). 24920856 10.1212/WNL.0000000000000592 PMC4117363

[42] Li, W. et al. Longitudinal functional connectivity changes related to dopaminergic decline in Parkinson’s disease. *Neuroimage Clin.* **28**, 102409 (2020). 32916466 10.1016/j.nicl.2020.102409 PMC7490914

[43] Mastro, K. J. et al. Cell-specific pallidal intervention induces long-lasting motor recovery in dopamine-depleted mice. *Nat. Neurosci.* **20**, 815–823 (2017). 28481350 10.1038/nn.4559 PMC5546121

[44] Assaf, F. & Schiller, Y. A chemogenetic approach for treating experimental Parkinson’s disease. *Mov. Disord.* **34**, 469–479 (2019). 30536778 10.1002/mds.27554

[45] Samuel, M. et al. Pallidotomy in Parkinson’s disease increases supplementary motor area and prefrontal activation during performance of volitional movements an H2(15)O PET study. *Brain* **120**, 1301–1313 (1997). 9278624 10.1093/brain/120.8.1301

[46] Grafton, S. T., Waters, C., Sutton, J., Lew, M. F. & Couldwell, W. Pallidotomy increases activity of motor association cortex in Parkinson’s disease: a positron emission tomographic study. *Ann. Neurol.* **37**, 776–783 (1995). 7778851 10.1002/ana.410370611

[47] Fukuda, M. et al. Functional correlates of pallidal stimulation for Parkinson’s disease. *Ann. Neurol.* **49**, 155–164 (2001). 11220735 10.1002/1531-8249(20010201)49:2<155::aid-ana35>3.0.co;2-9

[48] Festini, S. B. et al. Altered cerebellar connectivity in Parkinson’s patients ON and OFF L-DOPA medication. *Front. Hum. Neurosci*. **9**, 214 (2015). 10.3389/fnhum.2015.00214 PMC4405615 25954184

[49] Jech, R., Mueller, K., Schroeter, M. L. & Růžička, E. Levodopa increases functional connectivity in the cerebellum and brainstem in Parkinson’s disease. *Brain* **136**, e234 (2013). 23370091 10.1093/brain/awt015

[50] Zhong, M. et al. Effects of levodopa therapy on voxel-based degree centrality in Parkinson’s disease. *Brain Imaging Behav.* **13**, 1202–1219 (2019). 30091020 10.1007/s11682-018-9936-7

[51] Mottolese, C. et al. Mapping motor representations in the human cerebellum. *Brain* **136**, 330–342 (2013). 22945964 10.1093/brain/aws186

[52] O’Callaghan, C. et al. Cerebellar atrophy in Parkinson’s disease and its implication for network connectivity. *Brain* **139**, 845–855 (2016). 26794597 10.1093/brain/awv399

[53] Balsters, J. H., Laird, A. R., Fox, P. T. & Eickhoff, S. B. Bridging the gap between functional and anatomical features of cortico-cerebellar circuits using meta-analytic connectivity modeling. *Hum. Brain Mapp.* **35**, 3152 (2013). 24142505 10.1002/hbm.22392 PMC5293143

[54] Striemer, C. L., Cantelmi, D., Cusimano, M. D., Danckert, J. A. & Schweizer, T. A. Deficits in reflexive covert attention following cerebellar injury. *Front. Hum. Neurosci.* **9**, 428 (2015). 26300756 10.3389/fnhum.2015.00428 PMC4523795

[55] Armstrong, R. A. Visual Symptoms in Parkinson’s Disease. *Parkinson’s Dis.* **2011**, 908306 (2011). 21687773 10.4061/2011/908306 PMC3109513

[56] Rektorova, I., Krajcovicova, L., Marecek, R. & Mikl, M. Default mode network and extrastriate visual resting state network in patients with Parkinson’s Disease Dementia. *Neurodegenerative Dis.* **10**, 232–237 (2012). 10.1159/000334765 22269223

[57] Hannaway, N. et al. Visual dysfunction is a better predictor than retinal thickness for dementia in Parkinson’s disease. *J. Neurol. Neurosurg. Psychiatry* **94**, 742–750 (2023). 37080759 10.1136/jnnp-2023-331083 PMC10447370

[58] Disbrow, E. A. et al. Movement activation and inhibition in Parkinson’s disease: a functional imaging study. *J. Parkinson’s Dis.* **3**, 181 (2013). 23938347 10.3233/JPD-130181 PMC4586119

[59] Luo, C. et al. The trajectory of disturbed resting-state cerebral function in Parkinson’s disease at different Hoehn and Yahr stages. *Hum. Brain Mapp.* **36**, 3104–3116 (2015). 25959682 10.1002/hbm.22831 PMC6869419

[60] Cao, C. et al. L-dopa treatment increases oscillatory power in the motor cortex of Parkinson’s disease patients. *Neuroimage Clin.* **26**, 102255 (2020). 32361482 10.1016/j.nicl.2020.102255 PMC7195547

[61] Chung, J. W. et al. Beta-band oscillations in the supplementary motor cortex are modulated by levodopa and associated with functional activity in the basal ganglia. *NeuroImage Clin.* **19**, 559–571 (2018). 29984164 10.1016/j.nicl.2018.05.021 PMC6029579

[62] Shen, B. et al. Altered putamen and cerebellum connectivity among different subtypes of Parkinson’s disease. *CNS Neurosci. Ther.* **26**, 207–214 (2020). 31730272 10.1111/cns.13259 PMC6978269

[63] Rahimpour, S., Rajkumar, S. & Hallett, M. The supplementary motor complex in Parkinson’s disease. *J. Mov. Disord.* **15**, 21 (2021). 34814237 10.14802/jmd.21075 PMC8820882

[64] Blihar, D., Delgado, E., Buryak, M., Gonzalez, M. & Waechter, R. A systematic review of the neuroanatomy of dissociative identity disorder. *Eur. J. Trauma Dissociation* **4**, 100148 (2020). 10.1080/15299732.2020.1869650 33433297

[65] Dadario, N. B., Tanglay, O. & Sughrue, M. E. Deconvoluting human Brodmann area 8 based on its unique structural and functional connectivity. *Front. Neuroanat.* **17**, 1127143 (2023). 37426900 10.3389/fnana.2023.1127143 PMC10323427

[66] Kövari, E., Horvath, J. & Bouras, C. Neuropathology of Lewy body disorders. *Brain Res. Bull.* **80**, 203–210 (2009). 19576266 10.1016/j.brainresbull.2009.06.018

[67] Ng, B. et al. Distinct alterations in Parkinson’s medication-state and disease-state connectivity. *Neuroimage Clin.* **16**, 575–585 (2017). 28971008 10.1016/j.nicl.2017.09.004 PMC5608603

[68] Aleksovski, D., Miljkovic, D., Bravi, D. & Antonini, A. Disease progression in Parkinson subtypes: the PPMI dataset. *Neurol. Sci.* **39**, 1971–1976 (2018). 30109466 10.1007/s10072-018-3522-z

[69] Gonzalez-Robles, C. et al. Embedding patient input in outcome measures for long‐term disease‐modifying Parkinson disease trials. *Mov. Disord.* **39**, 433–438 (2024). 38140767 10.1002/mds.29691

[70] Fischl, B. & Dale, A. M. Measuring the thickness of the human cerebral cortex from magnetic resonance images. *Proc. Natl. Acad. Sci. USA* **97**, 11050–11055 (2000). 10984517 10.1073/pnas.200033797 PMC27146

[71] Fischl, B., Liu, A. & Dale, A. M. Automated manifold surgery: constructing geometrically accurate and topologically correct models of the human cerebral cortex. *IEEE Trans. Med. Imaging* **20**, 70–80 (2001). 11293693 10.1109/42.906426

[72] Destrieux, C., Fischl, B., Dale, A. & Halgren, E. Automatic parcellation of human cortical gyri and sulci using standard anatomical nomenclature. *Neuroimage* **53**, 1–15 (2010). 20547229 10.1016/j.neuroimage.2010.06.010 PMC2937159

[73] Fischl, B. et al. Whole brain segmentation: automated labeling of neuroanatomical structures in the human brain. *Neuron* **33**, 341–355 (2002). 11832223 10.1016/s0896-6273(02)00569-x

[74] Fischl, B. et al. Automatically parcellating the human cerebral cortex. *Cereb. Cortex* **14**, 11–22 (2004). 14654453 10.1093/cercor/bhg087

[75] Reuter, M. & Fischl, B. Avoiding asymmetry-induced bias in longitudinal image processing. *Neuroimage* **57**, 19–21 (2011). 21376812 10.1016/j.neuroimage.2011.02.076 PMC3260043

[76] Reuter, M., Schmansky, N. J., Rosas, H. D. & Fischl, B. Within-subject template estimation for unbiased longitudinal image analysis. *Neuroimage* **61**, 1402–1418 (2012). 22430496 10.1016/j.neuroimage.2012.02.084 PMC3389460

[77] Diedrichsen, J. A spatially unbiased atlas template of the human cerebellum. *NeuroImage* **33**, 127–138 (2006). 16904911 10.1016/j.neuroimage.2006.05.056

[78] Winkler, A. M., Ridgway, G. R., Webster, M. A., Smith, S. M. & Nichols, T. E. Permutation inference for the general linear model. *Neuroimage* **92**, 381–397 (2014). 24530839 10.1016/j.neuroimage.2014.01.060 PMC4010955

[79] Freedman, D. & Lane, D. A nonstochastic interpretation of reported significance levels. *J. Bus. Econ. Stat.* **1**, 292–298 (1983).

[80] Zalesky, A., Fornito, A. & Bullmore, E. T. Network-based statistic: identifying differences in brain networks. *Neuroimage* **53**, 1197–1207 (2010). 20600983 10.1016/j.neuroimage.2010.06.041

[81] Benjamini, Y. & Hochberg, Y. Controlling the false discovery rate: a practical and powerful approach to multiple testing. *J. R. Stat. Soc. Ser. B* **57**, 289–300 (1995).

[82] Jolliffe, I. T. *Principal Component Analysis*. 10.1007/b98835 (Springer-Verlag, 2002).

[83] Mwangi, B., Tian, T. S. & Soares, J. C. A review of feature reduction techniques in neuroimaging. *Neuroinformatics* **12**, 229–244 (2014). 24013948 10.1007/s12021-013-9204-3 PMC4040248

[84] Cortes, C. & Vapnik, V. Support-vector networks. *Mach. Learn* **20**, 273–297 (1995).

[85] Rubinov, M., Kötter, R., Hagmann, P. & Sporns, O. Brain connectivity toolbox: a collection of complex network measurements and brain connectivity datasets. *NeuroImage* **47**, S169 (2009).

