# Supplementary material for: Modulatory effect of levodopa on the basal ganglia-cerebellum connectivity in Parkinson’s disease
Source: NPJ Parkinsons Dis. 2025 May 6;11:115. doi: 10.1038/s41531-025-00954-9 (PMC12056079; doi:10.1038/s41531-025-00954-9)
Supplement: Supplementary file 1 — Supplementary Figure 1 [file 41531_2025_954_MOESM1_ESM.pdf]

Supplementary information

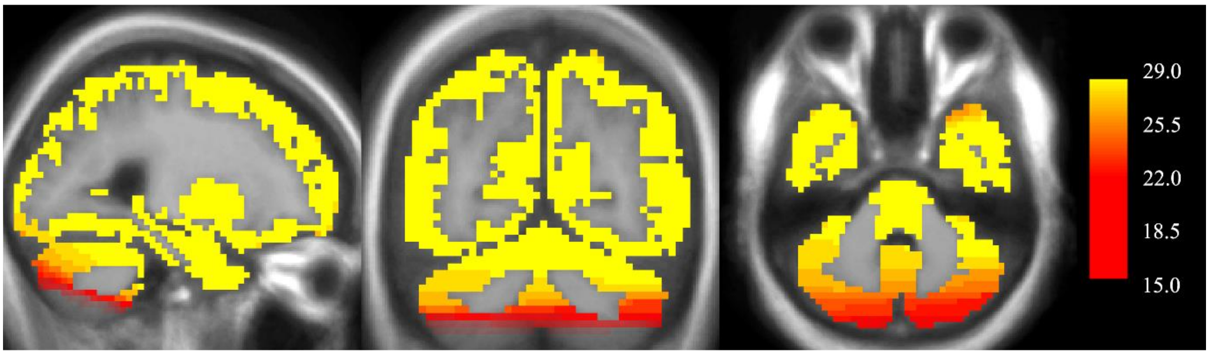

***Supplementary Figure 1:* Participants per voxel threshold.** The brain map shows the number of subjects per voxel with a threshold of 15 subjects applied. Voxels available in less than 15 subjects were excluded from the analysis. MNI coordinates of slice positions = (27, -65, -37).
